# Linking chemical data from the Comparative Toxicogenomics Database with adverse outcome pathways from the AOP-Wiki: a mechanistic data-oriented approach to help inform environmental health

**DOI:** 10.12688/f1000research.172567.1

**Published:** 2025-11-17

**Authors:** Allan Peter Davis, Thomas C. Wiegers, Daniela Sciaky, Fern Barkalow, Brent Wyatt, Jolene Wiegers, Roy McMorran, Sakib Abrar, Carolyn J. Mattingly

**Affiliations:** 1 Department of Biological Sciences, North Carolina Sate University, Raleigh, North Carolina, 27695, USA; 2 Center for Human Health and the Environment, North Carolina State University, Raleigh, North Carolina, 27695, USA

**Keywords:** environmental chemicals, adverse outcome pathways, autism, molecular mechanisms, database, disease networks, interoperability

## Abstract

**Background:**

Chemicals can perturb gene functions to affect chronic human diseases, and a significant amount of biological knowledge involved in environmental health is available in public databases. Combining information across resources can assist in the discovery of novel testable hypotheses related to how chemical exposures influence human diseases, such as autism.

**Methods:**

The Comparative Toxicogenomics Database (CTD) is a public resource that provides curated content for chemicals, genes, phenotypes, diseases, and exposures. The AOP-Wiki is a repository of adverse outcome pathways (AOPs) that provide defined biological frameworks describing disease processes. Here, we intersect CTD toxicogenomic content with the AOP-Wiki to identify environmental chemicals that could potentially modulate key steps in autism.

**Results:**

We identify numerous chemical stressors that intersect with the individual events of the autism AOP, including bisphenol compounds, per/polyfluoroalkyl substances, pesticides, metals, and air pollutants, suggesting a wide range of environmental factors that could synergize to potentially affect autism. By integrating additional CTD curated content for three autism-associated chemicals (bisphenol A, particulate matter, and valproic acid), we discover other mechanisms, including specific genes (e.g., SLC1A1, GSTP1, CNTNAP2) and phenotypes (e.g., lipid metabolism, inflammatory response, social behavior) that can be used to help refine or expand this AOP or create an entirely new pathway for autism. Furthermore, related diseases are identified to build interconnected networks, mechanistically linking autism to fatty liver disease, intellectual disability, and cancer.

**Conclusions:**

We demonstrate the value of integrating content from different resources to address environmental health questions related to autism etiology and co-morbidities. Importantly, our methodology is easily adapted for any AOP in the AOP-Wiki to identify potential environmental influences on the disease process and help support or refine AOPs. This analysis underscores the importance of standardizing public databases to make them efficiently interoperable for enhanced shared utility across the numerous bioknowledge digital landscapes.

## Introduction

Numerous public databases exist that provide extensive content related to chemicals, genes, phenotypes, biological networks, and diseases. ^1^ The shared use of standardized vocabularies facilitates data interoperability, integration, and exchange between these resources to help understand human health. ^2^ Synergy arising from combining information from different sources can lead to novel discoveries and testable hypotheses. Most chronic human diseases are the result of a complex, multifactorial interplay between environmental agents and genetics, ^3, 4^ and chemical exposure is an important element of the environment. Here, we integrate the content of two distinct publicly available databases to illuminate how environmental chemicals could affect autism, a human chronic disease influenced by numerous genes as well as environmental conditions. ^5^

For over two decades, the Comparative Toxicogenomics Database (CTD: https://ctdbase.org) has manually curated the scientific literature to annotate toxicogenomic information in a structured format using FAIR controlled vocabularies ( https://ctdbase.org/about/ctdDataFairness.jsp), providing detailed, contextualized interactions between chemicals, genes, phenotypes, anatomy terms, diseases, and exposures. ^6^ Currently, CTD includes over 4.1 million manually curated interactions describing relationships between 19,400 chemicals, 57,000 cross-species gene products, 6,900 phenotypes, 1,000 anatomical terms, and 7,200 diseases, curated from over 148,000 research papers ( https://ctdbase.org/about/dataStatus.go). In turn, CTD integrates these curated interactions to generate “Inference Networks”, predictive associations based upon shared intermediates ^7^ as well as “CGPD-tetramers”, modular four-unit blocks of information that prospectively relate an initiating chemical with an interacting gene and an intermediate phenotype to a disease outcome in a step-wise direction. ^8^ Importantly, CTD includes the original source articles as evidence for every curated statement used to generate all inferences and tetramers, providing transparency and traceability. CTD inferences and tetramers can be used to computationally fill the molecular knowledge gaps connecting chemical exposure to a variety of disease outcomes ^8– 10^ as well as group chemicals by mechanistic-induced adverse endpoints. ^11^

The AOP-Wiki ( https://aopwiki.org) is the official repository for a community-driven international development of adverse outcome pathways (AOPs), coordinated by the global policy forum Organization for Economic Co-operation and Development (OECD). ^12^ The AOP is a modular knowledge framework that relates intermediate key events (KE) by key event edge relationships (KER) at different levels of biological knowledge, connecting a molecular initiating event (MIE) to an adverse outcome (AO) to describe the critical check-points for a disease pathway. ^13^ AOPs can be used to organize and model biological knowledge as well as provide the necessary mechanistic information for new approach methodologies (NAMs) and integrated approaches to testing and assessment (IATA) for chemical risk analysis. ^13– 17^ Currently, the AOP-Wiki contains 532 AOPs in various stages of endorsement, the majority of which (74%) are flagged as “empty” with still no official endorsement, indicating a critical need to increase AOP testing, refinement, verification, and approval. Importantly, AOPs are defined as stressor-independent and chemical-agnostic, although they are typically reported with a list of assigned “prototypical stressors” that have been used to experimentally document key steps in the pathway and provide empirical support for the AOP, especially the KERs. ^18^ Independently, chemical stressors have been layered onto established AOPs to intertwine “stressor-AOP networks” that help inform chemical influence (both single and mixed chemical exposures) on interconnected disease pathways ^19^ and identify health outcomes associated with exposure to, for example, plastic additives ^20^ and inorganic cadmium. ^21^

Integrating toxicogenomic information from CTD with AOPs supplies the molecular mechanisms associated with chemical exposure to modulate biological pathways. Here, as a use case demonstration, we expand upon this concept of intersecting chemical data from CTD to the AOP-Wiki to identify and prioritize potential environmental influences for autism and discover additional mechanistic data that can be used to help support, inform, refine, and expand AOP development and interconnectivity. Importantly, these methods can be adapted for any AOP in the AOP-Wiki. This study underscores the importance of standardizing public databases to make them interoperable and increase their utility, and we discuss ways to further enhance data type connections between CTD and AOP-Wiki going forward.

## Methods

### CTD data version and analysis

Analysis was performed using CTD data available in September 2025 (revision 17923). CTD is updated with new content on a monthly basis ( https://ctdbase.org/about/dataStatus.go); consequently, query results described in this text may vary over time. For analysis, CTD data pages and query results were downloaded (available formats: CSV, Excel, XML, or TSV) into spreadsheets, and the sorting, advanced filtering, and subtotaling functions provided in Excel were used to survey and count the unique data types. The online tools Venny 2.1 ( https://bioinfogp.cnb.csic.es/tools/venny/index.html), and InteractiVenn ^22^ were used to compare and find shared data types.

### CTD data for autism and phenotypes

CTD uses the MEDIC disease hierarchy as a FAIR controlled vocabulary for annotating disease outcomes. ^23^ In MEDIC, “Autism Spectrum Disorder” (ASD; MESH:D000067877) is part of the mental disorder branch and is a parent to several descendant diseases, including “Autistic Disorder” (MESH:D001321), “Asperger Syndrome” (MESH:D020817), and “Adenylosuccinate lyase deficiency” (MESH:C538235); collectively, we refer to this set of diseases simply as “autism”. Since MEDIC is a hierarchy, all associated data (e.g., chemicals, genes, and phenotypes) annotated to descendant terms are subsumed and displayed to the parent term and can be retrieved by simply using ASD as the query term. All query results were combined and filtered to remove any duplicates arising from descendant terms. CTD operationally distinguishes “phenotypes” from “diseases”, wherein a phenotype is defined by a molecular, cellular, or physiological process term that does not exist in the MEDIC vocabulary (e.g., cell migration, retinoic acid receptor signaling pathway, apoptosis, regulation of blood pressure, etc.). To annotate chemical-induced phenotype data, CTD uses the Gene Ontology (GO) as a controlled FAIR vocabulary for phenotypes ^24^; thus, all CTD phenotypes are defined by GO terms and their GO accession identifiers (GO:ID).

### CTD term mapping to autism AOP events to derive intersecting chemicals

**Figure 1.**
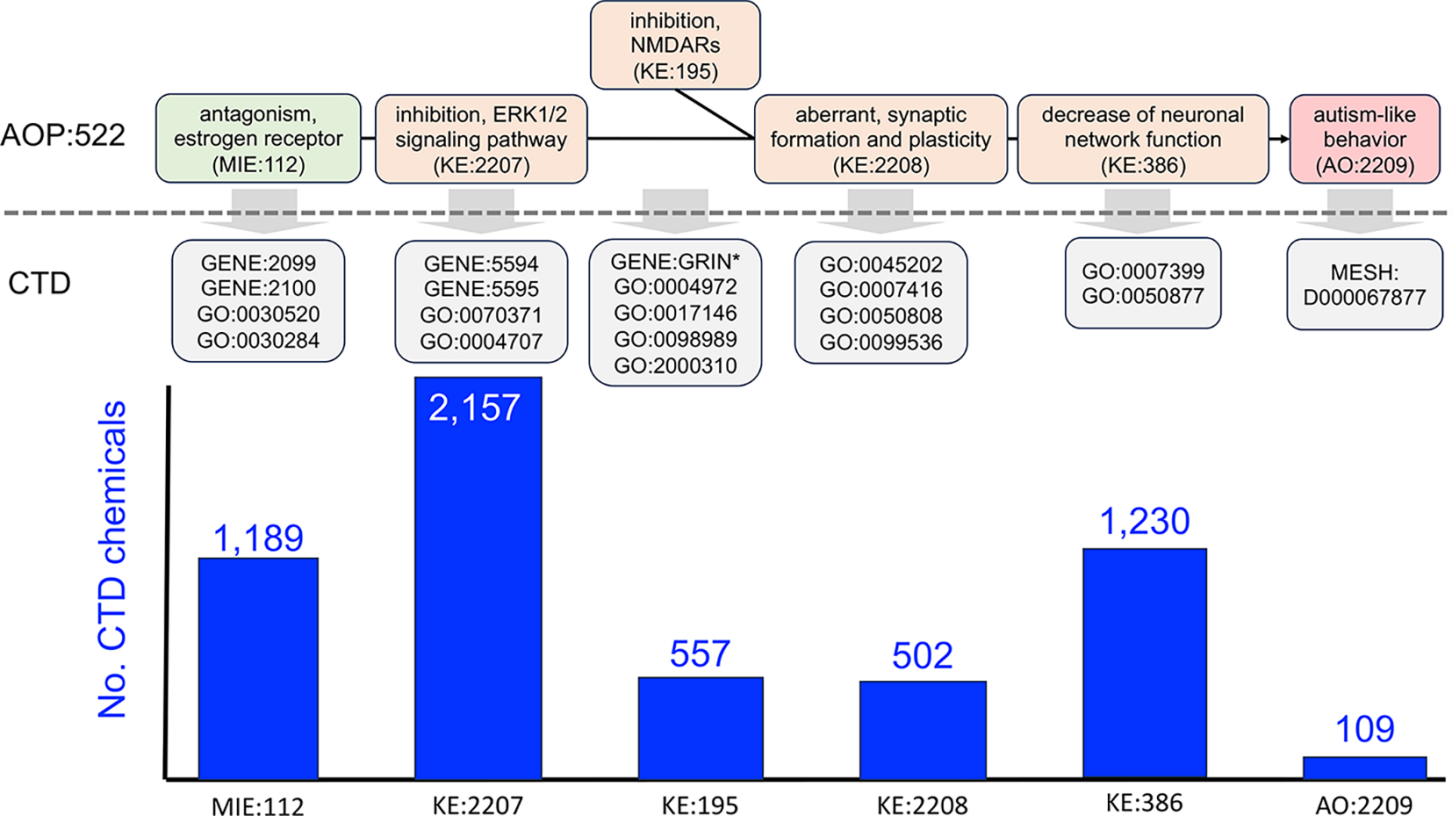
Mapping AOP:522 events to CTD mechanisms to find intersecting environmental chemicals from CTD. AOP:522 (“estrogen antagonism leading to increased risk of autism-like behavior”) is currently the only autism related pathway in the AOP-Wiki and is composed of one MIE with four KEs ending in the AO of autism-like behavior. KERs connecting KEs are represented as black lines. As represented in the AOP-Wiki, this AOP is bifurcated at the KE:195 event (“inhibition, NMDARs”, which at other times in the AOP-Wiki is entitled “decreased, NMDAR expression”). To enable interoperability, each AOP event is mapped to a corresponding CTD mechanistic data type, including a mix of CTD genes (GENE:IDs), phenotypes (GO:IDs), and the disease “Autism Spectrum Disorder” (MESH:ID). The CTD terms are described in the text ( Table 1) and are used to query and retrieve sets of CTD chemical stressors that can intersect each AOP event. In total, 3,648 unique chemicals are distributed across the six events of AOP:522, with the most numerous modulating the ERK1/ERK2 signaling pathway (KE:2207), followed by neuronal network function (KE:386) and estrogen receptor activity (MIE:112).

We searched the public AOP-Wiki database (version 2.7, 25 April 2025) with the term “autism” to retrieve AOP:522 ( https://aopwiki.org/aops/522) entitled “estrogen antagonism leading to increased risk of autism-like behavior” ( Figure 1). This AOP was last updated 25 January 2024 and is composed of six linked events: MIE:112 (“antagonism, estrogen receptor”), KE:2207 (“inhibition, ERK1/2 signaling pathway”), KE:195 (“inhibition, NMDARs” or “deceased, NMDAR expression”), KE:2208 (“aberrant, synaptic formation and plasticity”), KE:386 (“decrease of neuronal network function”), and AO:2209 (“autism-like behavior”). The edges connecting KE nodes to each other are referred to as key event relationships (KER). As currently structured in the AOP-Wiki, AOP:522 is bifurcated, with KE:195 as an offshoot event that joins the AOP at the KE:2208 junction ( Figure 1). We reviewed the individual AOP event terms to manually identify corresponding terms in CTD; sometimes the best match was to one or more genes and/or phenotypes ( Table 1). We used expansive queries when appropriate to fully cover the concepts best reflected in each KE; for example, we queried for chemicals that could affect any aspect of the estrogen receptor genes (gene expression, protein expression, protein activity, protein translocation, etc.). Since the GO is structured as a hierarchy, CTD queries with phenotype terms return data associated with the direct query term itself as well as associated data for all descendants of the phenotype term, providing comprehensive data coverage. Chemicals interacting with genes and phenotypes were retrieved using either CTD’s *Batch Query* tool ( https://ctdbase.org/tools/batchQuery.go), *Chemical-Gene Interaction Query* page ( https://ctdbase.org/query.go?type=ixn), or *Chemical-Phenotype Interaction Query* page ( https://ctdbase.org/query.go?type=phenotype). For each AOP event, CTD chemicals with directly curated interactions associated with the corresponding CTD genes/phenotypes were downloaded; duplicate listings derived from multiple queries were removed. For example, for MIE:112 (“antagonism, estrogen receptor”), we performed four independent CTD queries: first, we used the *CTD Chemical-Gene Interaction Query* tool to independently retrieve 1,088 and 603 chemicals that interacted with the genes for estrogen receptors 1 and 2 (ESR1, GENE:2099; ESR2, GENE:2100), respectively; next, we used the *CTD Chemical-Phenotype Interaction Query* tool to retrieve 180 chemicals that modulate the phenotype “intracellular estrogen receptor signaling pathway” (GO:0030520) and eight chemicals that modulate the phenotype “nuclear estrogen receptor activity” (GO:0030284); after compiling these four independent results and filtering out duplicates, we identified a set of 1,189 unique chemicals that modulate any one of the CTD mechanistic terms corresponding to AOP event MIE:112. This iterative process was performed for all six AOP events, using the corresponding CTD terms for queries ( Table 1) to derive 3,648 unique chemicals distributed over the six events comprising AOP:522 ( **Table S1** in Extended data ^25^). Chemicals were given a score of 1-6 based upon the number of AOP:522 events with which the chemical interacted, allowing the set to be ranked and prioritized for chemicals with the greatest intersection to AOP:522. Prioritized chemicals were group into categories by searching the CTD Chemical vocabulary ( https://ctdbase.org/voc.go?type=chem) for shared term parentage and/or using web-based searches for chemical definitions.

**Table 1.** CTD terms mapped to the six events of AOP:522 and the number of CTD chemicals annotated to each term.

AOP:ID	AOP term	CTD:ID	CTD term	No. CTD chemicals
MIE:112	Antagonism, estrogen receptor	GENE:2099	ESR1	1,088
		GENE:2100	ESR2	603
		GO:0030520	Intracellular estrogen receptor signaling pathway	180
		GO:0030284	Nuclear estrogen receptor activity	8
KE:2207	Inhibition, ERK1/2 signaling pathway	GENE:5594	MAPK1	2,045
		GENE:5595	MAPK3	2,018
		GO:0070371	ERK1 and ERK2 cascade	269
		GO:0004707	MAP kinase activity	34
KE:195	Inhibition, NMDARs or deceased, NMDAR expression	n/a	GRIN_wildcard	549
		GO:0004972	NMDA glutamate receptor activity	5
		GO:0017146	NMDA selective glutamate receptor complex	0
		GO:0098989	NMDA selective glutamate receptor signaling pathway	9
		GO:2000310	Regulation of NMDA receptor activity	13
KE:2208	Aberrant, synaptic formation and plasticity	GO:0045202	Synapse	25
		GO:0007416	Synapse assembly	25
		GO:0050808	Synapse organization	69
		GO:0099536	Synaptic signaling	450
KE:386	Decreased neuronal network function	GO:0007399	Nervous system development	620
		GO:0050877	Nervous system process	880
AO:2209	Autism-like behavior	MESH:D000067877	Autism Spectrum Disorder	109

n/a: Not applicable; Gene symbol query with wildcard and results manually vetted.

### Constructing a new AOP series for autism using CTD content

The *CTD Tetramers* tool ( https://ctdbase.org/query.go?type=tetramer) was used to retrieve computational tetramers via three independent queries with the chemical field set to bisphenol A (MESH:C006780), particulate matter (MESH:D052638), or valproic acid (MESH:D014635) and the disease field set to Autism Spectrum Disorder (MESH:D000067877), which also retrieves data for descendant disease terms. The resulting three datasets were manually combined in a spreadsheet and analyzed to identify the gene-phenotype pairs common to all three chemical-disease outputs, and this identified tetramer subset was uploaded to CTD’s *Chord Diagram Generator* tool ( https://ctdbase.org/tools/chord.go?window=upload) to visualize the information as a chord diagram and identify frequently used gene and phenotype components. ^26^ The phenotypes “response to oxidative stress” (GO:0006979), “glutathione metabolic process” (GO:0006749), “lipid metabolic process” (GO:0006629), “inflammatory response” (GO:0006954), “social behavior” (GO:0035176), and “locomotory behavior” (GO:0007626) were revealed as some of the most frequently used terms in the subset and were selected to manually construct a new AOP series. Since this dataset is derived from CTD tetramers, by operational definition, all three chemicals (bisphenol A, particulate matter, and valproic acid) have a direct interaction with all retrieved genes, phenotypes, and autism in CTD; similarly, all retrieved genes are directly annotated to every phenotype and directly curated to autism in CTD. The gene sets for each selected phenotype were manually collected and compared against each other to find subsets of shared genes that could be used to manually link the phenotypes.

### Finding autism-related diseases from shared CTD mechanisms

The *CTD Tetramers* tool was used to retrieve tetramers for independent queries for the 19 identified CTD gene or phenotype equivalent mechanistic terms that correspond to the first five events of AOP:522 ( Figure 1; Table 1): MIE:122 (GENE:2099, GENE:2100, GO:0030520, GO:0030284), KE:2207 (GENE:5594, GENE:5595, GO:0070371, GO:0004707), KE:195 (GENE:GRIN-wildcard, GO:0004972, GO:0017146, GO:00988989, GO:2000310), KE:2208 (GO:0045202, GO:0007416, GO:0050808, GO:0099536), and KE:386 (GO:0007399, GO:0050577). The tetramer results for each independent query were downloaded, compiled to identify a unique set of diseases for each of the five AOP events, and compared by Venn analysis. Each AOP event for AOP:522 was also queried in the AOP-Wiki to find other AOPs and AOs with which they were associated.

## Results and discussion

### Discovering environmental chemicals that can intersect with an autism AOP

As a use case, we queried the AOP-Wiki with the term “autism” to retrieve AOP:522 (“estrogen antagonism leading to increased risk of autism-like behavior”, https://aopwiki.org/aops/522). Currently, this AOP has the status of “empty” and is listed as “open for adoption”. The model was published in 2024 using public database content from numerous independent resources, including CTD, by looking for autism-related genes that also interact with two endocrine-disrupting chemicals, diethylhexyl phthalate (DEHP) and bisphenol A ^27^ as prototypical stressors. The model is composed of six events: one MIE, four KEs, and one AO ( Figure 1). We sought to leverage these six AOP events in order to expand upon the list of potentially associated chemicals beyond the original two endocrine disruptors. To identify chemicals in CTD that can intersect with these six AOP events, the AOP-Wiki terms were mapped to corresponding terms in CTD, and the selected CTD terms were used to retrieve sets of interacting chemicals from CTD ( Table 1), as such:

*MIE:112 (“antagonism, estrogen receptor*”). This MIE maps to four CTD data types, including two estrogen receptor genes ESR1 (GENE:2099) and ESR2 (GENE:2100) and two phenotypes “intracellular estrogen receptor signaling pathway” (GO:0030520) and “nuclear estrogen receptor activity” (GO:0030284). From this, we compiled a set of 1,189 unique CTD chemicals that have curated interactions with at least one of the four data types and can therefore affect MIE:112.

*KE:2207 (“inhibition, ERK1/2 signaling pathway”*). This KE maps to four CTD data types, composed of two mitogen-activated protein kinase genes MAPK1 (GENE:5594) and MAPK3 (GENE:5595) and two phenotypes “ERK1 and ERK2 cascade” (GO:0070371) and “MAP kinase activity” (GO:0004707). There are 2,157 unique chemicals curated in CTD that can affect KE:2207.

*KE:195 (“inhibition, NMDARs” or “deceased, NMDAR expression*”). This KE is described using two different names in the AOP-Wiki, referring to either the “inhibition” or “decreased expression” of N-methyl-D-aspartate receptors. Official nomenclature refers to these genes as “glutamate receptors” which use the gene symbol prefix of “GRIN” (e.g., GRIN1, GRIN2A, GRIN2B, etc.). To map this data type in CTD, we first queried for all chemical-gene interactions wherein the gene field was wildcarded using an asterisk (“GRIN*”) to maximize gene coverage. The downloaded interactions were then manually vetted to include only “GRIN”-based genes of which 23 were found, and the chemicals that interact with those 23 genes were collected. Additionally, this KE broadly maps to four CTD phenotypes, including: “NMDA glutamate receptor activity” (GO:0004972), “NMDA selective glutamate receptor complex” (GO:0017146), “NDMA selective glutamate receptor signaling pathway” (GO:0098989), and “regulation of NMDA receptor activity” (GO:2000310). In total, for all gene and phenotype queries, there are 557 unique CTD chemicals that can affect KE:195.

*KE:2208 (“aberrant, synaptic formation and plasticity*”). This KE maps to the four CTD phenotypes: “synapse” (GO:0045202), “synapse assembly” (GO:0007416), “synapse organization” (GO:0050808), and “synaptic signaling” (GO:0099536). There are 502 unique CTD chemicals that can affect KE:2208.

*KE:386 (“decreased neuronal network function*”). This KE broadly maps to the two CTD phenotypes “nervous system development” (GO:0007399) and “nervous system process” (GO:0050877) to include both neuronal development and function. There are 1,230 unique CTD chemicals that can affect KE:386.

*AO:2209 (“autism-like behavior*”). This AO maps to the CTD disease “Autism Spectrum Disorder” (ASD; MESH:D000067877), which is a parent term in the disease hierarchy and includes data for “Autistic Disorder” (MESH:D001321), “Asperger Syndrome” (MESH:D020817), and “Adenylosuccinate lyase deficiency” (MESH:C538235), a metabolic disease that presents with some symptoms of autism. We collectively refer to this set of disease terms as “autism”. To explore environmental influences on the etiology of autism, we used CTD chemicals annotated as “marker/mechanism” direct evidence to ASD (or one its descendants). There are 109 unique chemicals curated in CTD that can affect AO:2209.

**Figure 2.**
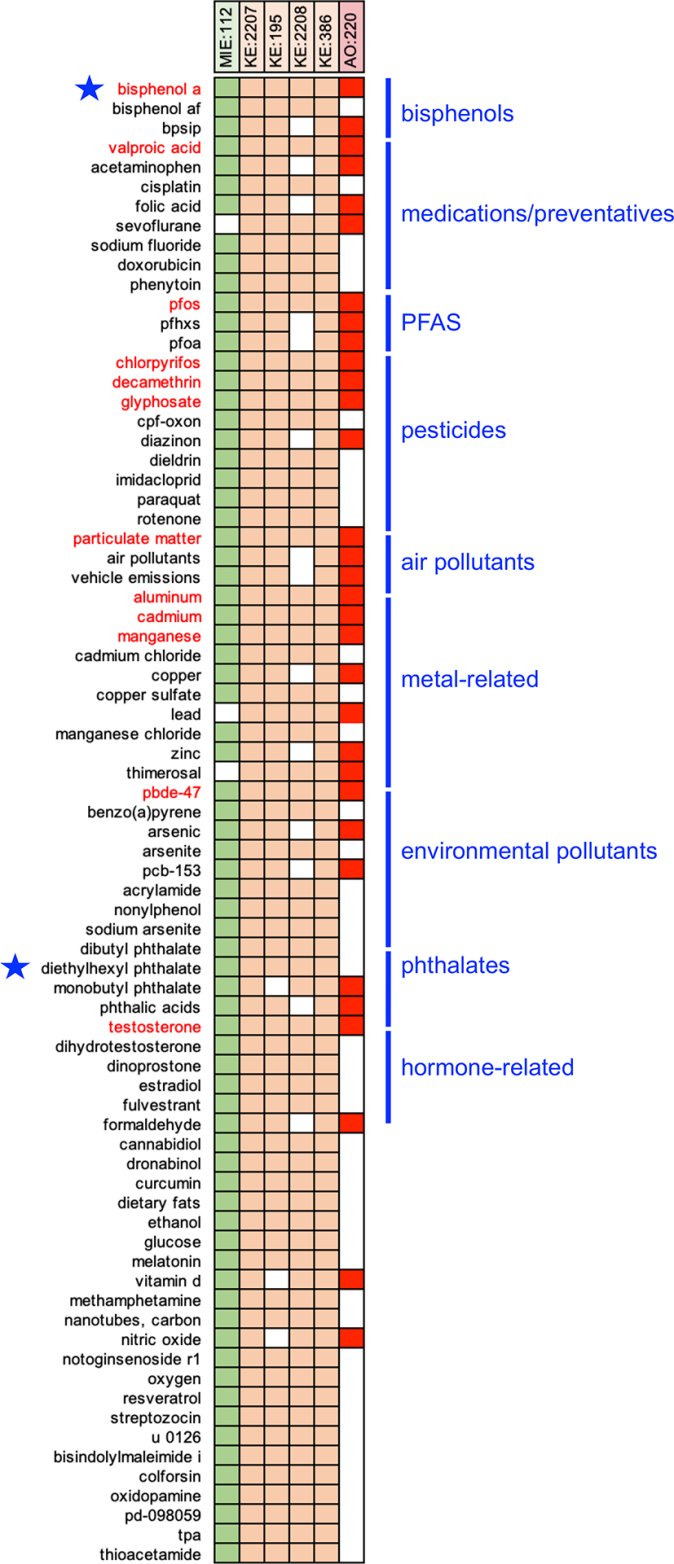
Prioritizing CTD chemical stressors for AOP:522. The 76 chemicals that interact with five or more of the six events in AOP:522 are depicted by a filled-in box showing the intersection between the chemical and the AOP events (listed at top). Twelve chemicals (red font) interact with all six AOP events. Many of these prioritized chemicals can be grouped into categories, including bisphenols, medications, PFAS, pesticides, air pollutants, metals, phthalates, etc. Bisphenol A and diethylhexyl phthalate (blue stars) are the prototypical stressors originally used to build AOP:522.

In total, 3,648 unique chemicals are distributed across the six events of AOP:522 ( Figure 1, and **Table S1** in Extended data ^25^). To help identify and prioritize the top environmental chemicals, we ranked the list by the number of individual AOP events with which each chemical interacts. Of the 3,648 chemicals, 76 modulate five or more of the AOP events ( Figure 2), and this set includes both bisphenol A and DEHP which are the original prototypical stressors used to construct AOP:522 in the AOP-Wiki ( https://aopwiki.org/aops/522#prototypical-stressors ). There are 12 chemicals that intersect with all six events of AOP:522, including bisphenol A, valproic acid, perfluorooctane sulfonic acid (PFOS), chlorpyrifos, decamethrin, glyphosate, particulate matter, aluminum, cadmium, manganese, 2,2’,4,4’-tetrabromodiphenyl ether (PBDE-47), and testosterone. Many of the 76 ranked chemicals that modulate five or more of the AOP events can be grouped as medications/preventatives (e.g., acetaminophen, valproic acid, folic acid, sodium fluoride), air pollutants, pesticides (e.g., paraquat, diazinon, imidacloprid), several per/polyfluoroalkyl substances (PFAS), metals (e.g., lead, copper, zinc), phthalates, and environmental pollutants ( Figure 2). These chemicals suggest that a wide range of environmental factors (independently or together) may modulate many of the key events currently included in AOP:522 to influence autism pathways. ^28^

Importantly, this approach of linking toxicogenomic content for environmental chemicals from CTD with AOPs provides molecular mechanisms for experiments and targeted assays to test modulation of the key elements of the AOP:522 pathway by any of the top-ranked environmental stressors to more definitively explore and confirm the possible influence of environmental factors on autism. This analysis is especially pertinent for studying the effects of co-exposed chemical mixtures, as environmental influences can be multifactorial. For example, low fluoride exposure induces autism-related neurotoxicity but only in the presence of aluminum cations, ^29^ and while prenatal exposure to either copper or butyl phthalates can be associated with depressive symptoms, only co-exposure to both has a significant association with autism. ^30^ Both air pollution and vehicle emissions are highly complex mixtures and have been linked to autism, ^31, 32^ but it becomes important to identify the specific compounds of those mixtures and evaluate them experimentally.

### Using environmental chemicals from CTD to discover additional events for autism AOP

Next, CTD environmental chemicals were leveraged to discover potential additional mechanisms that could expand AOP:522 (and fill in gaps) or generate completely new AOPs for autism. As a spectrum disease, autism exhibits a diverse and wide-ranging set of complex symptoms with different levels of severity affecting social interaction, communication, and behavior, suggesting the plausibility for a variety of biological pathways leading to an outcome. ^33^ We selected bisphenol A, the original prototypical stressor used to construct AOP:522 ^27^; valproic acid, an anti-convulsant drug whose use during pregnancy has been associated with autism in children ^34^ and is used experimentally to induce animal models of the disease ^35^; and particulate matter, a common element of air pollution, that has some evidence of association with autism from prenatal or early-life exposure. ^36^ All three of these chemicals additionally have curated exposure data in CTD correlating their exposure to autism in humans at the population level ( https://ctdbase.org/detail.go?type=disease&acc=MESH%3AD000067877&view=expStudies).

**Figure 3.**
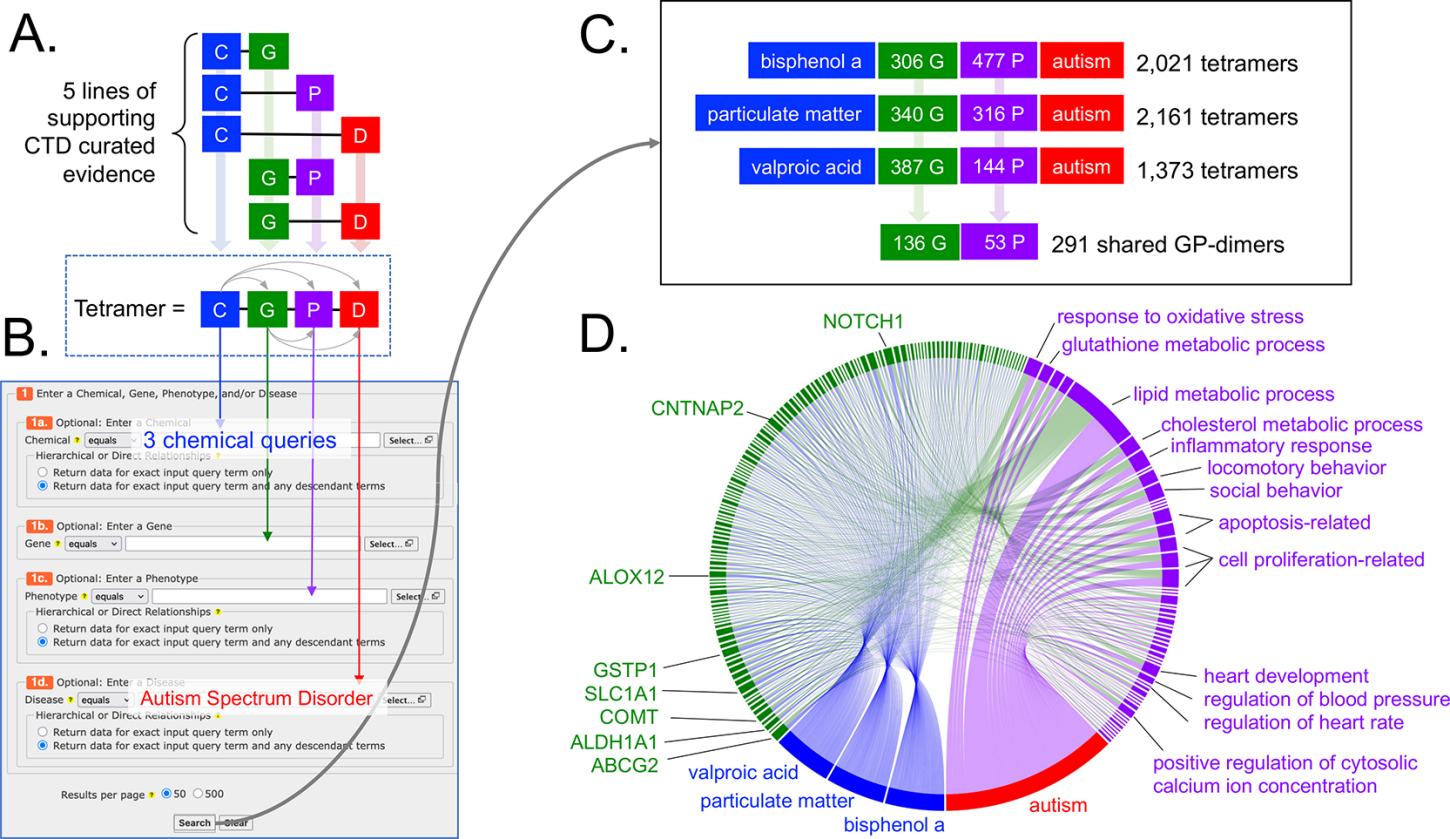
Leveraging CTD tetramers to find additional potential mechanisms for autism. (A) CTD tetramers are modular four-unit blocks of computed information linking an initiating chemical (C), an interacting gene (G), an intermediate phenotype (P), and a disease (D) outcome. To generate a tetramer, five lines of supporting evidence must already exist in CTD. Importantly, if a tetramer is generated, then, by operational definition, the chemical must have a directly curated relationship to the gene, phenotype, and disease in CTD, and every gene must be annotated to the phenotype and disease in CTD (dotted box, with five small arrows connecting all four blocks of the tetramer). (B) The online tool *CTD Tetramers* allows users to easily and quickly generate tetramers for any chemical, gene, phenotype, and/or disease of interest, by filling in any one or more of the four appropriate fields on the query page. Here, three independent queries were made for three chemicals as the input (bisphenol A, particulate matter, and valproic acid) with ASD as the disease output (which retrieves data for both ASD and AD) for each query. (C) Results include 2,021 tetramers for bisphenol A (using 306 genes and 477 phenotypes), 2,161 tetramers for particulate matter (using 340 genes and 316 phenotypes), and 1,373 tetramers for valproic acid (using 387 genes and 144 phenotypes). From these three tetramer sets, 291 shared GP-dimers are found for all three chemicals linked to autism, and involve 136 genes and 53 phenotypes. (D) The subset of tetramers representing only the shared 291 GP-dimers are visualized as a chord diagram, linking the three initiating chemicals (blue nodes), 136 genes (green nodes), and 53 phenotypes (purple nodes) to autism (red node, wherein data for ASD and AD are depicted as a single node), to illuminate potentially key mechanisms for consideration to refine or expand autism disease pathways.

We looked for additional molecular/cellular events that might help inform or refine AOP:522 by identifying mechanisms (i.e., genes and phenotypes) used by bisphenol A, particulate matter, and valproic acid in relationship to autism in CTD in an effort to find shared processes. We used the *CTD Tetramers* tool, ^37^ a user-friendly online tool that quickly generates computational solutions called CGPD-tetramers that are derived by integrating five curated supporting lines of literature-based evidence from CTD to construct a modular four-unit block linking an initiating chemical, an interactive gene, an intermediate phenotype event, and a disease outcome ( Figure 3A). Three independent queries were made using bisphenol A, particulate matter, and valproic acid as the chemical inputs and ASD as the disease output to retrieve 2,021, 2,161, and 1,373 computational tetramers, respectively ( Figure 3B-C, and **Tables S2-S4** in Extended data ^25^). The three sets of tetramers share 291 gene-phenotype paired intermediates (GP-dimers), composed of 136 genes and 53 phenotypes, that relate the three chemicals to autism, providing new potential key mechanisms to be included in a disease pathway. Some of the top shared genes underlying connections between all three chemicals and autism ( Figure 3D) include those that play a role as environmental sensors/detoxifiers (e.g., ABCG2, GSTP1, ALDH1A1) or function in neural health (e.g., SLC1A1, ALOX12, CNTNAP2, COMT, NOTCH1), helping to link environmental responses and neuronal phenotypes to autism. The most frequent phenotype shared by all three chemicals related to autism is lipid metabolism ( Figure 3D). Additional highlighted phenotypes include regulation of cell population proliferation and/or apoptosis, inflammatory response, oxidative stress, social and locomotory behavior, glutathione metabolism, cholesterol metabolism, heart development, heart rate, blood pressure, and cytosolic calcium ion concentration ( Figure 3D).

**Figure 4.**
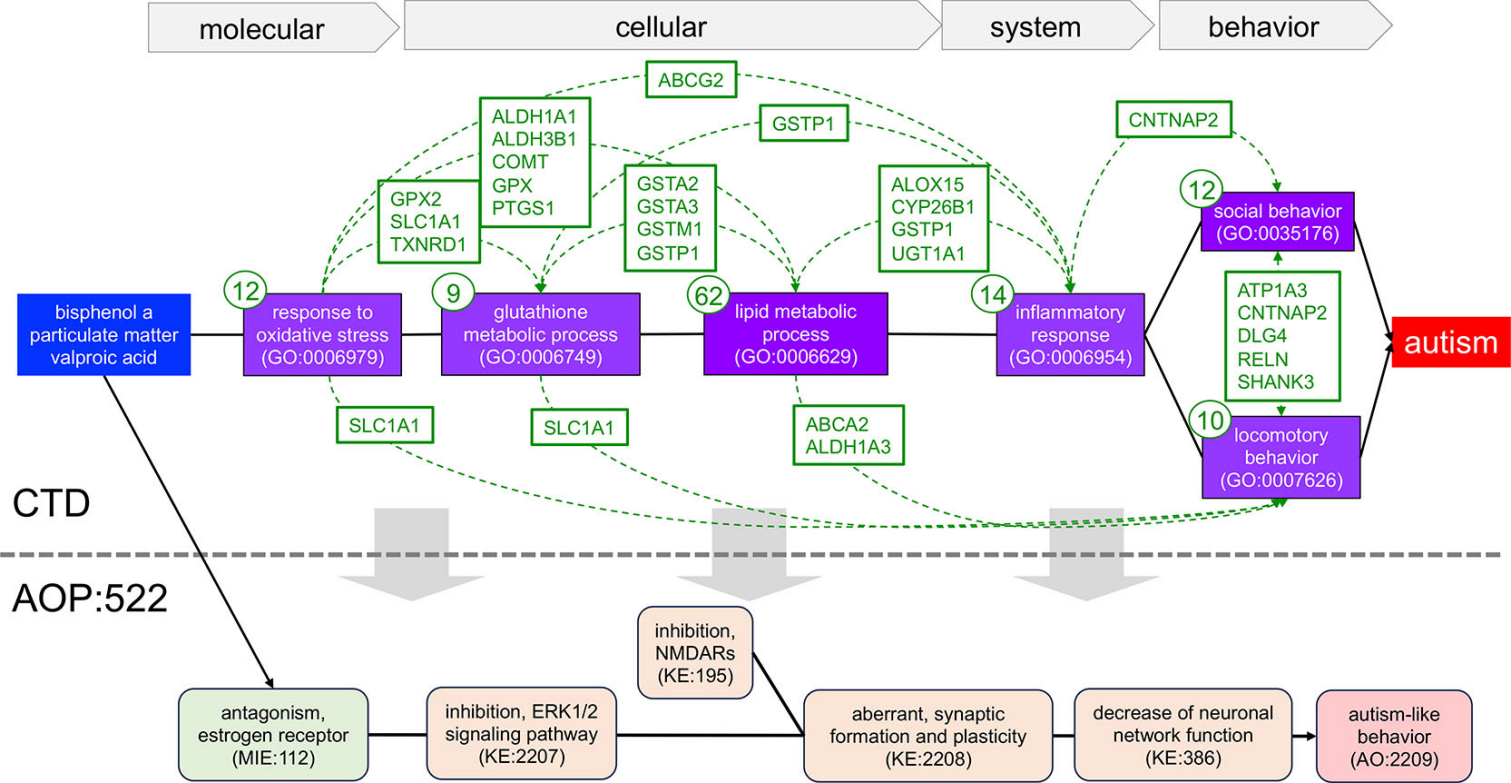
Constructing a new AOP series identified by CTD environmental chemicals associated with autism. We used 93 genes and six phenotypes identified as part of the shared GP-dimers from tetramers linking bisphenol A, particulate matter, and valproic acid to autism in CTD to manually build an intricately connected, novel AOP series for autism, spanning molecular, cellular, system, and behavioral phenotypes (purple boxes with GO:IDs). Since data are derived from CTD tetramers (see Figure 3A), by definition, all three chemical stressors (blue box) have directly curated relationships to every gene, phenotype, and autism in CTD (not shown), and every gene also has a directly curated relationship to autism (not shown) and is annotated to their associated phenotypes (gene number in small circle for each phenotype). Genes shared between any two phenotypes (boxes with listed gene symbols connected by dotted arcs) provide additional mechanistic links further supporting the numerous KERs between the events. This novel, manually generated AOP can serve as a framework to construct an entirely new AOP for autism or be used to refine or expand (gray downward arrows) AOP:522 from the AOP-Wiki (bottom).

We selected six of these prominent tetramer-identified key phenotypes and their associated 93 genes to manually construct a prospective pathway of linked events relating the three chemical stressors to autism ( Figure 4). In this strategy, CTD tetramers provide the literature-based content to build a highly interconnected mechanistic map that includes chemicals and genes directly curated to autism, genes and phenotypes directly curated to the three chemical stressors, and overlapping genes shared between the phenotypes as mechanistic links connecting the key events. Here, an oxidative stress response is linked to cellular metabolism (affecting the levels of glutathione and lipids, such as cholesterol), inflammatory responses, and both social and locomotor behavior. The edge relationships between each phenotype are further supported by shared genes ( Figure 4), which interact with each of the three chemicals and independently have curated relationships to autism. This novel AOP series has multiple levels of evidentiary support from CTD for the relationship edges connecting the phenotype events, and is supported in the extrinsic literature, wherein the role of oxidative stress, glutathione metabolism, impaired lipid metabolism, inflammatory processes, and both social and locomotory behaviors have all been found to promote the pathogenesis of or be a characteristic of autism ^38– 43^; as well, non-autistic-related associations connect oxidative stress, dietary lipids/cholesterol, and inflammation with changes in social behavior. ^44– 46^ Importantly, this new proposed AOP series computed from CTD tetramers can be used to create an entirely new pathway for autism or help refine and/or expand the current AOP:522 ( Figure 4), as well as provide new mechanisms to develop additional targeted assays. ^47^

### Using CTD to discover related diseases to build interconnected networks for autism

One of the hallmarks of AOPs is that the individual events are modular and can be re-used in other AOPs, ^48^ similar to the way CTD tetramers are modular and the underlying chemicals, genes, phenotypes, and diseases can be used in other tetramers. ^8^ This modular nature makes it possible to interrelate other AOPs (and CTD tetramers) that use the same data types. Thus, extrinsic diseases linked to the same corresponding CTD terms for the AOP:522 events can be connected to autism via their shared mechanisms.

**Figure 5.**
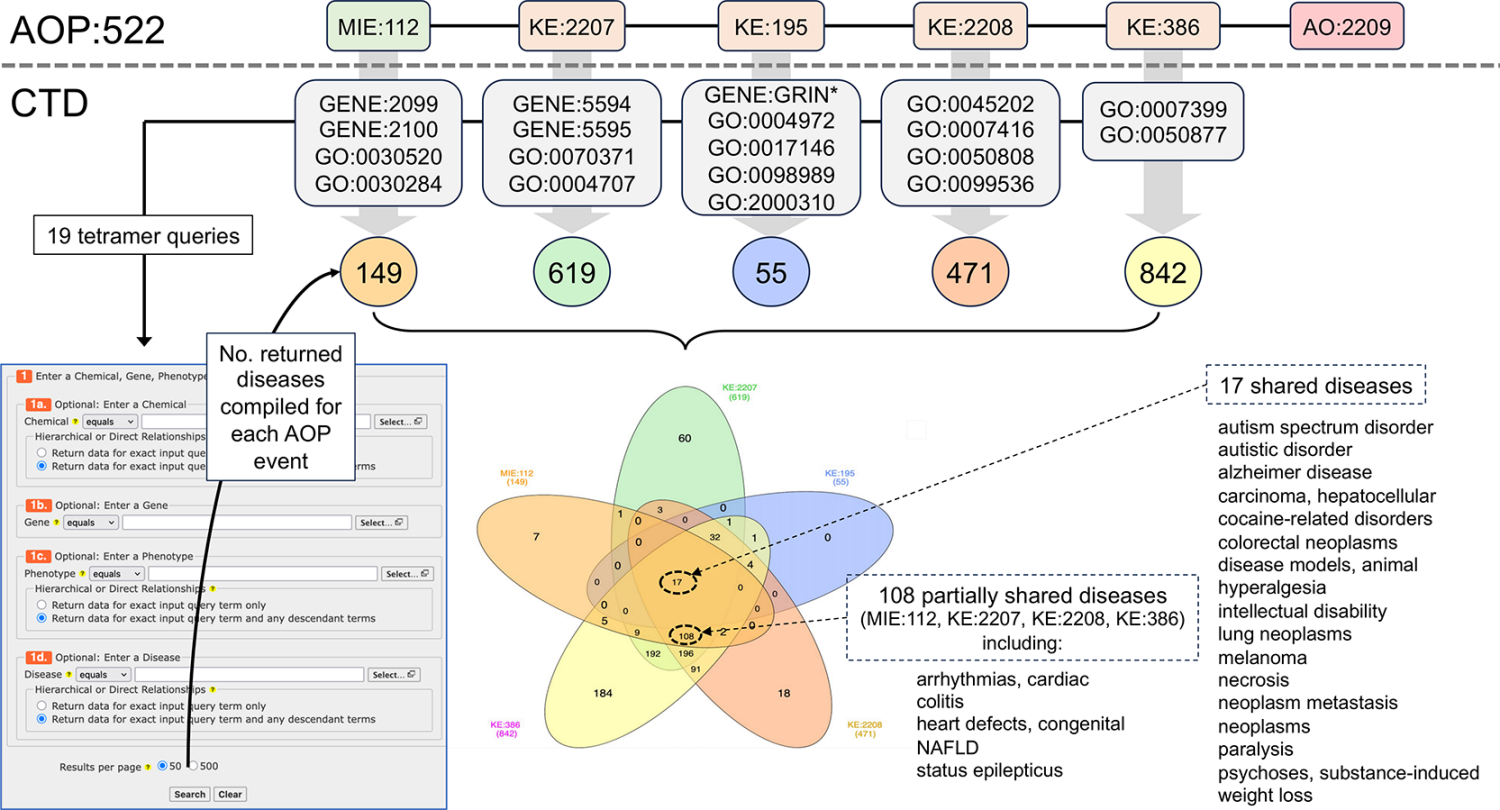
Discovering CTD diseases related to autism via shared intermediate mechanistic events. The first five AOP:522 events map to 19 corresponding CTD gene and phenotype terms (gray boxes with GENE:IDs and GO:IDs; also see Table 1). These CTD terms are independently used as query inputs in the *CTD Tetramers* tool to retrieve the tetramer-associated diseases compiled for each AOP mechanistic step. Sets of 149, 619, 55, 471, and 842 tetramer-derived CTD diseases are linked to MIE:112, KE:2207, KE:195, KE:2208, and KE:386, respectively (for simplicity, the AOP is drawn linear, and not bifurcated). A Venn analysis identifies 17 shared diseases simultaneously associated with all five AOP events. If KE:195 (the KE with the lowest number of diseases) is dropped from the analysis, an additional 108 shared diseases are identified (partial list shown) for the remaining four AOP events together (MIE:112, KE:2207, KE:2208, and KE:386).

Here, we use the same CTD gene and phenotype terms that were previously mapped to the first five AOP events of MIE:112, KE:2207, KE:195, KE:2208, and KE:386 ( Figure 1, Table 1), and we utilize the *CTD Tetramers* tool to retrieve and compile 149, 619, 55, 471, and 842 tetramer-derived diseases, respectively, for each of the five events for AOP:522 ( Figure 5). When the five datasets are analyzed, 17 shared diseases are identified that can be simultaneously associated with all five AOP events. Both ASD and AD are in this subset and also included are a variety of cancer-related outcomes, hyperalgesia, and intellectual disability, which have been reported with autism. ^49– 51^ If KE:195 (“inhibition, NMDARs”), which yielded the fewest number of tetramer-derived diseases and is represented as the bifurcated step in the original AOP:522, is excluded from the analysis, an additional 108 shared diseases are identified that co-use just the four remaining events MIE:112, KE:2207, KE:2208, and KE:386. These additional diseases include cardiac arrhythmias, congenital heart defects, non-alcoholic fatty liver disease (NAFLD), colitis, and status epilepticus, all of which have been associated with autism. ^52– 56^

**Figure 6.**
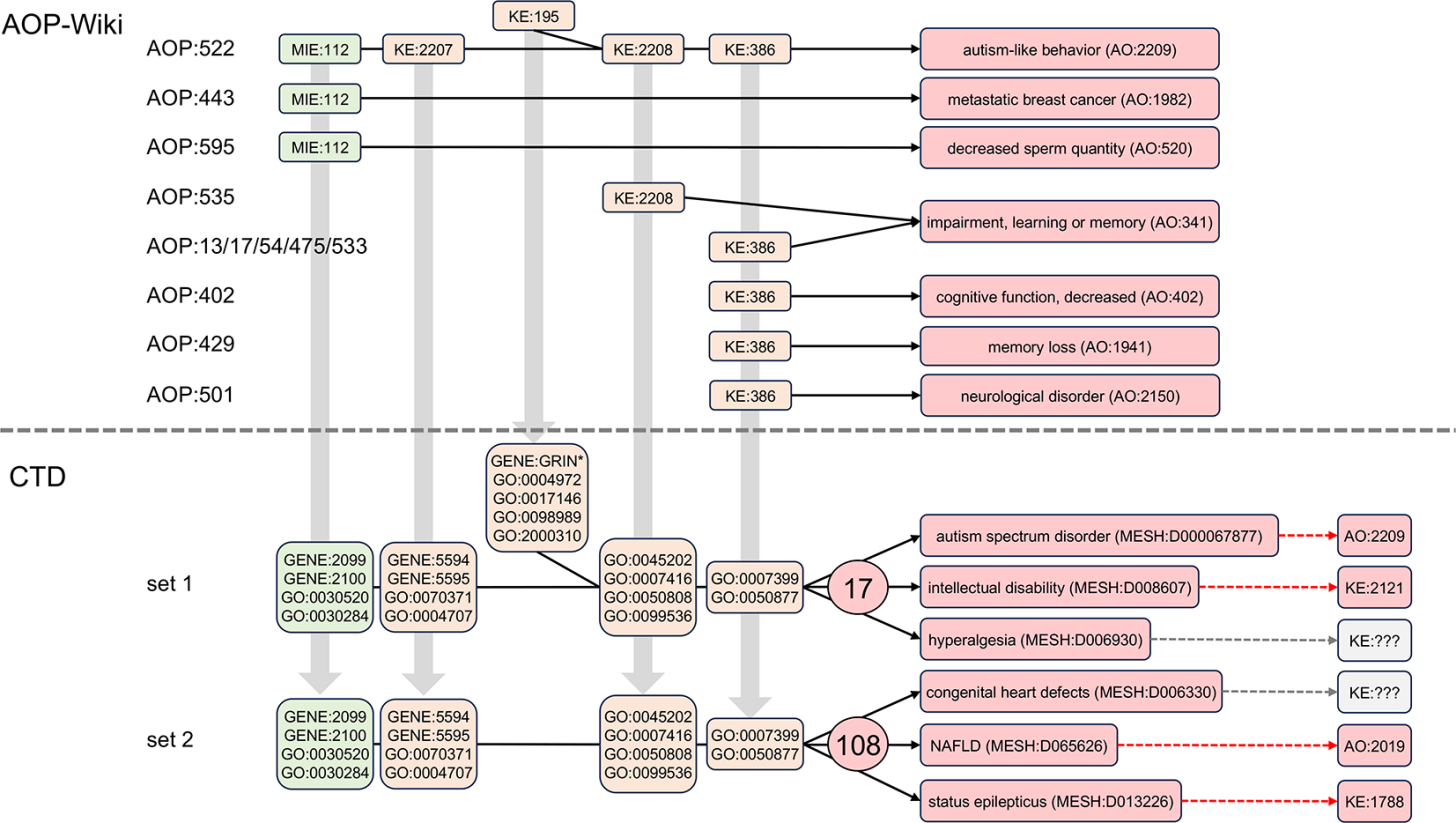
Building interconnected disease networks via shared CTD mechanisms. Five linked events composing AOP:522 result in autism-like behavior (AO:2209). Some of these individual modular events, however, are also used in other AOPs (but not all in conjunction with each other) resulting in other outcomes, such as MIE:112 ending in “metastatic breast cancer” (AO:1982) in AOP:443 or in “decreased sperm quantity” (AO:520) for the unrelated AOP:595. Both KE:2208 and KE:386 are involved in “impairment, learning or memory” (AO:341), but individually via numerous different AOPs, and KE:386 also leads to a related outcome of “memory loss” (AO:1941), yet by another AOP:429. Five of the AOP events, however, when mapped into their corresponding CTD mechanistic terms, and in simultaneous conjunction with each other (set 1), can be leveraged to discover 17 shared diseases that interconnect autism with numerous other health outcomes, such as intellectual disability and hyperalgesia. If KE:195 (the bifurcated event) is removed from analysis (set 2), an additional 108 shared diseases are identified, including congenital heart defects, non-alcoholic fatty liver disease (NAFLD), and status epilepticus. Many of these computed outcomes currently exist in the AOP-Wiki as AO or KE terms (dashed arrows).

Disease pathways that include the same modular events can be interconnected ( Figure 6), which may inform possible comorbidities for autism. To date, there are no AOPs in the AOP-Wiki that use all five of the same events used in the AOP:522 autism pathway, but individual events are included in a few limited AOPs. The initiating event MIE:112 (“antagonism, estrogen receptor”) is also used in AOP:443 leading to “metastatic breast cancer” (AO:1982) and independently in AOP:595 resulting in “decreased sperm quantity” (AO:520). As well, both KE:2208 (“aberrant, synaptic formation and plasticity”) and KE:386 (“decrease of neuronal network function”) are also part of two independent AOPs ending in “impairment, learning or memory” (AO:341), and KE:386 is additionally used in other AOPs resulting in the similar outcomes of “cognitive function, decreased” (AO:402) and “memory loss” (AO:1941).

CTD, on the other hand, discovers many more potential connected disease pathways that, importantly, include all five AOP events concurrently: MIE:112, KE:2207, KE:195, KE:2208, and KE:386 ( Figure 5), allowing additional mechanistic and interrelated disease networks to be constructed. Many of these computed outcomes have corresponding KE and AO terms in the AOP-Wiki to help construct AOP networks ( Figure 6).

### Limitations and strengths

A limitation to this study is that the AOP-Wiki does not impose controlled vocabularies or standardized formatting when new MIEs, KEs, or AOs are created and submitted by researchers. ^17^ Thus, to intersect CTD chemical content with AOPs, KE terms from the AOP-Wiki first must be manually mapped to corresponding terms in CTD, which is rarely a straightforward process, as some KE terms can be mapped to multiple phenotypes as well as genes, such as KE:2207 (“inhibition of ERK1/2 signaling pathway”) mapped here to both a CTD biological phenotype (“ERK1 and ERK2 cascade”, GO:0070371) as well as specific CTD target genes MAPK1 (GENE:5594) and MAPK3 (GENE:5595) whose activity can be modulated by chemicals. Additionally, many KE terms are incompletely described in the AOP-Wiki, such as KE:195, which sometimes is referred to as “decreased NMDAR expression” and other times as “inhibition, NMDARs”. To accommodate for this subtlety, we mapped KE:195 to CTD mechanistic terms reflecting both NMDAR gene expression as well as NMDAR activity. Bias can be introduced in the manual mapping of AOP-Wiki terms to matched mechanisms in CTD; for example, what exactly do the AOP authors mean by the KE term “decreased neuronal network function” (KE:386)? Here, the KE term is not defined in the AOP-Wiki, so it is left to each user to interpret this event, as we did here to refer to any impaired development (GO:0007399) or processes (GO:0050877) of the nervous system. To help initiate interoperability, the AOP-Wiki does provide a rudimentary download file ( https://aopwiki.org/downloads/aop_ke_ec.tsv) that maps many (but not all) AOP event terms to some GO terms (as well as other vocabularies), but the source of this mapping is unclear, as well as how the mapping was performed; nonetheless, it can serve as an important initial guide to start linking AOP event terms with CTD terms via shared GO terms. Additionally, some biomedical data translators ^57^ may be able to assist in rendering mappings between KE and GO terms impartially.

In 2022, CTD manually processed and mapped the then-current AOs from the AOP-Wiki to equivalent CTD disease or phenotype terms ^37^; however, over time, AO terms have been edited and new ones have been added to the AOP-Wiki, making the CTD mapping outdated. Other methods have been developed to increase the usability of AOP information, such as converting the AOP-Wiki into semantic web formats ^58^ or curating relevant KEs to gene sets associated with pathways, phenotypes, and GO terms ^59^; but these deliverables are catered mostly toward users proficient in programming. Finally, the AOP-Wiki does provide a list of more than 840 prototypical stressors currently used in their AOPs ( https://aopwiki.org/stressors), and these stressors are defined using common and stable chemical identifiers, including Chemical Abstract Service (CAS) numbers, Distributed Structure-Searchable Toxicity Identifiers (DTXSIDs), and International Chemical Identifier (InChI) keys, all of which are also used to define chemicals at CTD, providing an accessible integration point between these resources with respect to chemoinformatics. ^60^ Enhancing the AOP-Wiki with a dedicated team of professional biocurators could help to ensure data standardization and harmonization. Furthermore, requiring AOP contributors to include suggested FAIR controlled terms with their initial submissions, ^17^ especially defining KEs with suitable GO terms (a commonly used ontology in bioinformatics), ^61^ would be a significant step in opening up AOP data to more biological databases and researchers.

Lastly, we note a caveat about relying solely on CTD tetramers as a source of mechanistic data for building new AOPs and discovering interconnected disease networks. CTD tetramers require five lines of literature-based evidence for the constituent, curated interactions between a chemical, gene, phenotype, and disease ( Figure 3A). If any one of the five curated statements does not exist in CTD, the tetramer will not be generated. Thus, tetramers represent a more restrictive subset of computational solutions. As a counterpoint, the strength to tetramers is that they provide a more detailed and comprehensive view of outcome pathways than inferred relationships, which do not include all four data types. Importantly, CTD is not a static resource, as new literature is curated and added on a monthly basis, and the number of available tetramers increases over time. Furthermore, tetramers now can be prioritized and ranked by their calculated weighted “evidence strength score” to enable users to sort tetramers by the number of underlying articles from which mechanistic events were originally curated. ^6^ To complement tetramers, the less-restrictive predictions generated from CTD “Inference Networks” can also be used in modeling.

### Future directions

CTD is exploring new avenues to better enable the intersection of both CTD content and CTD tools with AOP datasets, such as an automated process to electronically transform CTD tetramer query results into mechanistic-linked maps connected by shared chemical and gene edges (see Figure 4); this is especially important in that the KERs connecting individual KEs in an AOP are often described as the critical core unit in supporting and advancing the success of an AOP. ^62^ As well, if AOP terms become controlled and stabilized, direct hyperlinks and accession interoperability between the shared data types of these two resources will advance accessibility and reusability, ^17^ similar to how CTD currently provides links and interoperable searches for chemicals to both PubChem ^63^ and CompTox, ^64^ genes to NCBI-Gene, ^65^ phenotypes to AmiGO, ^66^ anatomy terms to both Uberon ^67^ and Cell Ontology, ^68^ and diseases to Disease Ontology. ^69^

## Conclusion

We describe a method to explore environmental health issues by intersecting toxicogenomic chemical data from CTD with AOPs from the AOP-Wiki to show how public resources can be leveraged to discover new information about disease pathways. Here, we present autism as a use case in our analysis, but the same methodology can be adapted for any AOP in the AOP-Wiki. More than 3,600 chemical stressors are identified that could potentially influence AOP:522 for autism; of these, 76 chemicals can be prioritized (because they intersect with a preponderance of the AOP events), including medications/preventatives, air pollutants, pesticides, PFAS compounds, metals, phthalates, and environmental pollutants, suggesting a wide-range of environmental factors with the potential to influence autism etiologies and outcomes. These identified chemicals further discover additional environmental sensor and neural health genes as well as oxidative stress and metabolic, inflammatory, and behavioral mechanisms for consideration to expand or refine AOP:522 or to generate a new disease pathway for autism. Finally, additional diseases that use the same intermediate mechanisms discerned by CTD can be interconnected to build extensive comorbidity networks for autism. Importantly, CTD provides the supporting literature used to generate these testable mechanistic pathways. Leveraging this information to improve and refine AOP construction and validation may facilitate the process of official endorsement and status advancement for AOPs. This work underscores the importance of harmonizing public databases to increase their interoperability and utility across the bioknowledge landscape.

## Data Availability

All CTD content and analysis tools are freely available for non-commercial users at https://ctdbase.org. Figshare: Environmental chemicals from the Comparative Toxicogenomics Database linked to autism disease pathways. https://doi.org/10.6084/m9.figshare.30384805.v1 ^25^ The project contains the following extended datasets:
1.
Table S1. xlsx (CTD environmental chemicals distributed across six events of AOP:522 for autism). 2.
Table S2. xlsx (CTD tetramers for bisphenol A and Autism Spectrum Disorder). 3.
Table S3. xlsx (CTD tetramers for particulate matter and Autism Spectrum Disorder). 4.
Table S4. xlsx (CTD tetramers for valproic acid and Autism Spectrum Disorder). Extended data are available under the terms of the Creative Commons Attribution 4.0 International license (CC-BY 4.0).
