# Supplementary material for: Linking chemical data from the Comparative Toxicogenomics Database with adverse outcome pathways from the AOP-Wiki: a mechanistic data-oriented approach to help inform environmental health
Source: F1000Res. 2026 Apr 27;14:1266. Originally published 2025 Nov 17. [Version 2] doi: 10.12688/f1000research.172567.2 (PMC13231133; doi:10.12688/f1000research.172567.2)
Supplement: Peer review history

# Reviewer report: Reviewer response for version 2

**DOI:** 10.5256/f1000research.198672.r479505

**Published:** 2026-06-02

**Authors:** Brian N Chorley

**Affiliations:** 1 US Environmental Protection Agency, Research Triangle Park, North Carolina, USA

Authors satisfied all of my concerns. Thank you!

Is the work clearly and accurately presented and does it cite the current literature?

Yes

If applicable, is the statistical analysis and its interpretation appropriate?

I cannot comment. A qualified statistician is required.

Are all the source data underlying the results available to ensure full reproducibility?

Yes

Is the study design appropriate and is the work technically sound?

Yes

Are the conclusions drawn adequately supported by the results?

Yes

Are sufficient details of methods and analysis provided to allow replication by others?

Yes

Reviewer Expertise:

molecular biology, biomarkers, AOPs

I confirm that I have read this submission and believe that I have an appropriate level of expertise to confirm that it is of an acceptable scientific standard.

# Reviewer report: Reviewer response for version 1

**DOI:** 10.5256/f1000research.190304.r467900

**Published:** 2026-04-07

**Authors:** Brian N Chorley

**Affiliations:** 1 US Environmental Protection Agency, Research Triangle Park, North Carolina, USA

This manuscript by Davis et al. provides a case study to leverage the Comparative Toxicogenomics Database (CTD) to provide chemical context and/or supplemental content for Adverse Outcome Pathways (AOPs) in the AOP-Wiki. The use case here in an AOP associated with autism, specifically AOP:522 "Estrogen antagonism leading to an increased risk of autism-like behavior". Two approaches leveraging the CTD were outlines:  First, 6 KEs of AOP:522 were associated with GO and MESH terms, linking to chemicals using CTD search tools. Second, "CTD Tetramers" generated chemical-gene-phenotype-disease blocks associated with autism linked exposures (BPA, valproic acid, and PM) to derive literature-driven AOPs to both enhance existing AOPs (such as AOP:522) or create new ones for hypothesis generation, testing, or tool building.

 The manuscript is well-written, logically arranged, easy to follow and enhanced with helpful figures. The outlined procedures provide a theoretical blueprint to enhance existing AOPs, or generate new AOPS, using the continuously updated content of the CTD. Therefore, this manuscript suggests a path to fill in numerous gaps for existing “empty” AOPs, as well creating new content for AOs of interest. The steps provided, however, entail many manual steps that may be somewhat subjective and result in a daunting number of results. I have some suggestions, primarily minor, that should enhance the content of this manuscript.

**From Methods/CTD term mapping to autism AOP events to derive intersecting chemicals**: In Table 1, add a column that explicitly states which CTD search tool (Chemical-Gene Interaction Query or Chemical-Phenotype Interaction Query) was used to map to CTD chemicals. Although it may be obvious, this clearly links the process to the results listed.

**From Methods/Constructing a new AOP series for autism using CTD content**: “The gene sets for each selected phenotype were manually collected and compared against each other to find subsets of shared genes that could be used to manually link the phenotypes.” It is not clear what was done here. Were the greater number of shared genes selected to link to phenotypes or were frequent subsets of genes selected? Please explain in more detail.

**From Methods/Finding autism-related diseases from shared CTD mechanisms**: “Each AOP event for AOP:522 was also queried in the AOP-Wiki to find other AOPs and AOs with which they were associated.” I would like to see a complete list of AOs associated

 with these events other than those selected in Fig 6. Can this be made as a supplement?

**From Results/Using environmental chemicals from CTD to discover additional events for autism AOP**: “We selected six of these prominent tetramer-identified key phenotypes…” Mark these somehow in Fig 3D for easy reference. Also, it isn’t clear why these six specifically were selected. Please better describe the selection process used.

 “Importantly, this new proposed AOP series computed from CTD tetramers can be used to create an entirely new pathway for autism or help refine and/or expand the current AOP:522 (Figure 4), as well as provide new mechanisms to develop additional targeted

 assays.” It is not clear how this new AOP helps define AOP:522. Suggest examples here or simply only state this is a new AOP.

**Figure 5**: Suggest using an UpSet plot rather than a complex Venn. This provides an easier-to-follow comparison.

Is the work clearly and accurately presented and does it cite the current literature?

Yes

If applicable, is the statistical analysis and its interpretation appropriate?

I cannot comment. A qualified statistician is required.

Are all the source data underlying the results available to ensure full reproducibility?

Yes

Is the study design appropriate and is the work technically sound?

Yes

Are the conclusions drawn adequately supported by the results?

Yes

Are sufficient details of methods and analysis provided to allow replication by others?

Yes

Reviewer Expertise:

molecular biology, biomarkers, AOPs

I confirm that I have read this submission and believe that I have an appropriate level of expertise to confirm that it is of an acceptable scientific standard, however I have significant reservations, as outlined above.

# Response

**Published:** 2026-04-14

**Authors:** Allan Davis

**Affiliations:** CTD, USA

**1. From Methods/CTD term mapping to autism AOP events to derive intersecting chemicals: **In Table 1, add a column that explicitly states which CTD search tool (Chemical-Gene Interaction Query or Chemical-Phenotype Interaction Query) was used to map to CTD chemicals. Although it may be obvious, this clearly links the process to the results listed.

**AUTHORS’ RESPONSE**:  There are several different ways to retrieve CTD chemicals that intersect with the mapped AOP steps, including the Chemical-Gene Interaction Query (for chemicals interacting with mapped gene terms), the Chemical-Phenotype Interaction Query (for chemicals interacting with mapped phenotype terms), or the CTD Batch Query (for chemicals interacting with either mapped genes or mapped phenotypes).  As well, users can simply go to the respective CTD gene page or CTD phenotype page and download the chemical set from the “Chemical” data-tab from each page.  We think modifying Table 1 with a new column that simply repeats “Chemical-Gene Interaction Query” for each gene term and “Chemical-Phenotype Interaction Query” for each phenotype term would be distracting. Instead, in Supplementary Figure S2 we now demonstrate how to retrieve these chemical sets with comprehensive step-by-step instructions using the CTD Batch Query and include a detailed diagram as well.  We also have made this distinction (between gene and phenotype queries) clearer in the “Methods” section with added text.

**2. From Methods/Constructing a new AOP series for autism using CTD content**: “The gene sets for each selected phenotype were manually collected and compared against each other to find subsets of shared genes that could be used to manually link the phenotypes.” It is not clear what was done here. Were the greater number of shared genes selected to link to phenotypes or were frequent subsets of genes selected? Please explain in more detail.

**AUTHORS’ RESPONSE**:  We have now added a detailed Supplementary Figure S4 (figshare) that describes step-by-step instructions on how to construct this new AOP series, including identifying the tetramer genes that are shared between different tetramer phenotypes, and how these shared gene edges can be used to connect adjacent phenotypes and help design testable hypotheses to find supporting KER evidence.

**3. From Methods/Finding autism-related diseases from shared CTD mechanisms**: “Each AOP event for AOP:522 was also queried in the AOP-Wiki to find other AOPs and AOs with which they were associated.” I would like to see a complete list of AOs associated with these events other than those selected in Fig 6. Can this be made as a supplement?

**AUTHORS’ RESPONSE**:  A complete list of the AOPs and AOs associated with the five MIE/KEs of AOP:522 is now provided as Supplementary Figure S6 (figshare).

**4. From Results/Using environmental chemicals from CTD to discover additional events for autism AOP**: “We selected six of these prominent tetramer-identified key phenotypes…” Mark these somehow in Fig 3D for easy reference. Also, it isn’t clear why these six specifically were selected. Please better describe the selection process used.

**AUTHORS’ RESPONSE**:  We selected six of the most prominent phenotypes illuminated in the chord diagram of Figure 3D (now identified in that figure with asterisks).  We have added expanded text for clarification in the “Results”. As well, we have added a new Supplementary Figure S4 to show the step-by-step instructions for this process (figshare).

**5. “**Importantly, this new proposed AOP series computed from CTD tetramers can be used to create an entirely new pathway for autism or help refine and/or expand the current AOP:522 (Figure 4), as well as provide new mechanisms to develop additional targeted assays.” It is not clear how this new AOP helps define AOP:522. Suggest examples here or simply only state this is a new AOP.

**AUTHORS’ RESPONSE**:  Our intention by that statement was to suggest that users could “refine and/or expand” AOP:522 by inclusion of some of the additional mechanistic key events derived from CTD tetramers.  This is also suggested in Figure 4, which states in the legend: “This novel, manually generated AOP can serve as a framework to construct an entirely new AOP for autism or be used to refine or expand (gray downward arrows) AOP:522 from the AOP-Wiki.”

**6.** **Figure 5**: Suggest using an UpSet plot rather than a complex Venn. This provides an easier-to-follow comparison.

**AUTHORS’ RESPONSE**:  We admit we have never heard of (nor seen) an UpSet plot before, and we feel that a casual reader also will not be familiar with how to read/interpret one.  Hence, we prefer to keep our original Venn diagram in the full text, but we have since learned how to create an UpSet plot and now include it as an alternative visualization in new Supplementary Figure S5 (figshare), and point users to that alternative plot in both the text and Figure 5 legend.

# Reviewer report: Reviewer response for version 1

**DOI:** 10.5256/f1000research.190304.r462004

**Published:** 2026-03-26

**Authors:** Jason Obrien

**Affiliations:** 1 Environment and Climate Change Canada, Ottawa, Canada

**Study Summary**

- Data from the Comparative Toxicogenomic Database were linked to an Adverse Outcome Pathway from the AOPwiki to identify potentially useful chemical, gene, phenotype, disease and AOP relationships. The resulting relationships are potentially useful for identifying exposures that may influence the apical adverse outcome, or provide mechanistic support for the AOP.
- The authors selected AOP 522 (“Estrogen antagonism leading to increased risk of autism-like behavior”) as a pilot case study, but the approach could in theory be applied to any AOP.
- The authors identified >3000 chemicals in the CTD database that have some relationship to one or more of the Key Events (KEs) in AOP 522, 76 of which had relationships with five or more of the AOPs six KEs.
- Of these 76 chemicals, the authors selected three (Bisphenol A, valproic acid and particulate matter) to identify 136 genes and 53 phenotypes related to these chemicals and autism.
- The authors propose that the identified genes and phenotypes can form the basis of additional KEs related to the AOP, or be used to provide mechanistic support existing KEs. To demonstrate this, the authors selected 6 of the resulting phenotypes to construct a hypothetical AOP linking oxidative stress to autism.
- Finally, the authors used the CTD database to identify several other diseases that may have mechanistic overlap with AOP 522.

**Evaluation Summary**

 Overall, this manuscript describes a well-conceived and executed study. The introduction presents clear and concise background information, with clear and justified objectives. The methods are clearly described and reproducible (I was even successful at replicating some of the CTD search results). The results are clearly conveyed and adequately discussed. I have only a few minor comments below for the author’s consideration.

**Specific Comments**

**Comment regarding Methods section: **There are a lot of “results” presented in the methods section. For example, the number of chemicals returned from queries is reported on page 6, paragraph 1; the most frequent phenotypes were reported on page 7, paragraph 2. This is not a major problem, per se, but it is a bit unconventional.

**Comment regarding selected AOP: **The AOP selected, AOP:522, is a very underdeveloped AOP. Although the authors do allude to this in their discussion (the AOP is “empty” and “open for adoption”) I suggest that this point can be made more clear and maybe earlier in the paper (for example in the intro). For example, this could be emphasized more in the last paragraph of the introduction (e.g. We identified intersecting data between CTD and AOPwiki to reveal chemical, gene, phenotype relationships that could potentially be used as supporting evidence to strengthen an autism-related AOP that is in the early stages of development.)

**Comment regarding KE:386 mapping: **The CTD terms selected for this KE do not seem very well matched. The KE is called “decreased neuronal network function”, and based on the description in AOPwiki, the KE is largely related to measured synaptic activity. The CTD terms are “nervous system development” and “nervous system process”, which seem more general than what the KE describes. The authors do address this later in the limitations section, but might be better to mention in the mapping section.

**Comment 1 regarding support for AOP: **The authors state that their results “provide molecular mechanisms for experiments and targeted assays to test modulation of the key elements of AOP:522”. I completely agree with this, but feel that this point can be elaborated on a bit. Specifically, these results can be used to identity experiments in the literature or design new experiments that can be used as supporting evidence (for example KER evidence) to further develop and strengthen this AOP.

**Comment 2 regarding support for AOP: **The authors propose that the identified relationships can be used to “expand AOP development and interconnectivity”. The authors nicely show how these results can be used to identify potentially new KEs or create AOP networks. However, one of the greatest challenges in AOP development is providing support for the Key Event Relationships (KERs). While the authors do mention this briefly in the future directions section, it would have been nice to see an example of how the CTD results can be integrated using AOP theory to provide empirical support for the KERs.

**Comment regarding hypothetical AOP: **How did the authors select the six phenotypes from the 53 common phenotypes to include in their prospective AOP? Similarly, how did the authors select the order of the phenotypes in the prospective AOP? Were these decisions data driven, or based on expert knowledge?

**Minor Typo**: Page 7, paragraph 1: Prioritized chemicals were group **ED** into categories

Is the work clearly and accurately presented and does it cite the current literature?

Yes

If applicable, is the statistical analysis and its interpretation appropriate?

Not applicable

Are all the source data underlying the results available to ensure full reproducibility?

Yes

Is the study design appropriate and is the work technically sound?

Yes

Are the conclusions drawn adequately supported by the results?

Yes

Are sufficient details of methods and analysis provided to allow replication by others?

Yes

Reviewer Expertise:

NA

I confirm that I have read this submission and believe that I have an appropriate level of expertise to confirm that it is of an acceptable scientific standard, however I have significant reservations, as outlined above.

# Response

**Published:** 2026-04-14

**Authors:** Allan Davis

**Affiliations:** CTD, USA

**1. Comment regarding Methods section: **There are a lot of “results” presented in the methods section. For example, the number of chemicals returned from queries is reported on page 6, paragraph 1; the most frequent phenotypes were reported on page 7, paragraph 2. This is not a major problem, per se, but it is a bit unconventional.

**AUTHORS’ RESPONSE**:  We have edited the “Methods” section to make it more generic and instead moved the specific “results” to the more appropriate “Results” section of the manuscript.

**2. Comment regarding selected AOP**: The AOP selected, AOP:522, is a very underdeveloped AOP. Although the authors do allude to this in their discussion (the AOP is “empty” and “open for adoption”) I suggest that this point can be made more clear and maybe earlier in the paper (for example in the intro). For example, this could be emphasized more in the last paragraph of the introduction (e.g. We identified intersecting data between CTD and AOPwiki to reveal chemical, gene, phenotype relationships that could potentially be used as supporting evidence to strengthen an autism-related AOP that is in the early stages of development.)

**AUTHORS’ RESPONSE**:  In response to similar comments from Reviewer 1, we have edited the last paragraph of the “Introduction” to now more clearly explain that our test AOP is underdeveloped, open for adoption, and currently contains limited data, and that by intersecting CTD content with this AOP, users can discover chemicals, genes, and phenotypes that can help develop and strengthen this AOP.

**3. Comment regarding KE:386 mapping**: The CTD terms selected for this KE do not seem very well matched. The KE is called “decreased neuronal network function”, and based on the description in AOPwiki, the KE is largely related to measured synaptic activity. The CTD terms are “nervous system development” and “nervous system process”, which seem more general than what the KE describes. The authors do address this later in the limitations section, but might be better to mention in the mapping section.

**AUTHORS’ RESPONSE**:  As the reviewer notes, we believe a limitation in this study is the mapping of KE terms (often weakly defined or susceptible to interpretation) from the AOP-Wiki to their best corresponding matches in CTD.  We discuss this in the “Results” section for KE:195 (which is sometimes described as “NMDAR inhibition” and at other times as “decreased NMDAR expression” in the AOP-Wiki).

 For AOP:522, KE:2208 (“aberrant, synaptic formation and plasticity”) is immediately upstream to KE:386 (“decrease of neuronal network function”).  However, KE:2208 is poorly defined in the AOP-Wiki (with only a key event title and no other context) while the downstream KE:386 is almost overly defined with extensive descriptions in different sections of the webpage (ranging from the measurement of synaptic activity to neuron and brain development).  This makes it challenging to interpret the best mapping for these two supposedly distinct KEs.  To further highlight this issue, we have added two new passages to the “Results”:

 “Mapping terms across databases, however, is not always straightforward and can be subject to interpretation, and individual users can exercise flexibility to arrive at different translations, especially with respect to the level of granularity.  In the AOP-Wiki, KE terms are defined in multiple sections, including the “event title” (a mandatory descriptive title for the KE), the “event component” (an optional ontology term, if known or applicable), and an “event description” (an optional descriptive passage about the biological state being measured and its role in biology).  We attempted to balance these descriptions for each unique KE to make the best mappings to CTD.”

 and then later:

 “While KE:386 uses a broad event title (“decreased neuronal network function”), its event component is more nuanced (“decreased synaptic signaling”), which conceptually overlaps with the upstream key event KE:2208 (see above).  However, the event description for KE:386 details the neuronal network processes in the developing and mature brain.  To limit duplicative mapping used for KE:2208 (“synaptic signaling” GO:0099536), and, more importantly, to cover the essential features of neuron and brain development, we decided to map this KE more broadly to two CTD phenotypes: “nervous system development” (GO:0007399) and “nervous system process” (GO:0050877) which includes “neurogenesis” (GO:0022008), “brain development” (GO:0007420), and “transmission of nerve impulse” (GO:0019226), among many other neuronal functions and processes”.

**4. Comment 1 regarding support for AOP**: The authors state that their results “provide molecular mechanisms for experiments and targeted assays to test modulation of the key elements of AOP:522”. I completely agree with this, but feel that this point can be elaborated on a bit. Specifically, these results can be used to identity experiments in the literature or design new experiments that can be used as supporting evidence (for example KER evidence) to further develop and strengthen this AOP.

**AUTHORS’ RESPONSE**:  We have now added text to elaborate this point in the “Results”: “Linking literature-based chemical-gene-phenotype events (curated from mechanistic studies) into biologically plausible pathways can support KERs as well as provide testable hypotheses for additional experiments to quantify empirical evidence connecting these intermediate events in response to the same chemical stressor and via shared mechanistic genes (25).”

**5. Comment 2 regarding support for AOP**: The authors propose that the identified relationships can be used to “expand AOP development and interconnectivity”. The authors nicely show how these results can be used to identify potentially new KEs or create AOP networks. However, one of the greatest challenges in AOP development is providing support for the Key Event Relationships (KERs). While the authors do mention this briefly in the future directions section, it would have been nice to see an example of how the CTD results can be integrated using AOP theory to provide empirical support for the KERs.

**AUTHORS’ RESPONSE**:  Using CTD tetramers to discover new potential intermediate mechanistic phenotypes/KEs and construct new AOP series creates a highly interconnected map of linked phenotypes/KEs that have numerous edges via shared chemical-gene, chemical-phenotype, and gene-phenotype relationships (see Figure 4); this offers curated literature-based support for KERs as well as new avenues for developing additional experiments to further validate the KERs.  Also, results from CTD Tetramers Query are ranked by an “Evidence Strength Score”, calculated as the product score of the number of references used in each of the four lines of supporting evidence needed to construct a CGPD-tetramer.  Thus, computational tetramers with a higher number of source articles across the five supporting lines of evidence categories will have a higher calculated “Evidence Strength Score” and be ranked at the top of the output results, providing users with an option to filter tetramers with a higher amount of underlying literature support; this is also diagrammed as step 4 in the newly added Supplementary Figure S3.  We have added to the description of our new model AOP constructed from shared tetramer data, describing how these identified tetramers provide “overlapping genes shared between the phenotypes as mechanistic links connecting the key events and provide support for KER evidence”.  This is also currently described in the Figure 4 legend: “Genes shared between any two phenotypes (boxes with listed gene symbols connected by dotted arcs) provide additional mechanistic links further supporting the numerous KERs between the events”.

**6. Comment regarding hypothetical AOP**: How did the authors select the six phenotypes from the 53 common phenotypes to include in their prospective AOP? Similarly, how did the authors select the order of the phenotypes in the prospective AOP? Were these decisions data driven, or based on expert knowledge?

**AUTHORS’ RESPONSE**:  We selected six of the most prominent phenotypes illuminated in the chord diagram of Figure 3D (now identified in that figure with asterisks, per Reviewer 3) and manually ordered them at four levels of biological organization.  We have added expanded text for clarification in the “Results”. As well, we have added a new Supplementary Figure S4 to show the step-by-step instructions for this process.

**7. Minor Typo**: Page 7, paragraph 1: Prioritized chemicals were groupED into categories

**AUTHORS’ RESPONSE**:  We have fixed this typo.

# Reviewer report: Reviewer response for version 1

**DOI:** 10.5256/f1000research.190304.r456179

**Published:** 2026-03-05

**Authors:** Holly M Mortensen

**Affiliations:** 1 US Environmental Protection Agency, Research Triangle Park, North Carolina, USA

- **Issue with selected AOP -Status  “open for adoption”**

AOP 522 is classified as "open for adoption" and is poorly populated in the AOP Wiki

 Cui et al: Previous study has shown that prenatal or postnatal exposure to EEDs could increase the risk of ASD in children (Mandy and Lai, 2016).

 Mandy and Lai, 2016 clearly state that  "Although the studies generally showed a positive association between EDCs and ASD, after considering the strengths and limitations, we concluded that the **overall strength of evidence supporting an association between prenatal exposure to EDCs and later ASD in humans remains "limited" and inconclusive. **Further well-conducted prospective studies are warranted to clarify the role of EDCs on ASD development."

- **Manuscript would benefit from an analysis workflow. **

The authors present a complicated analysis routine that is difficult to follow. Individual steps are described in sufficient detail, but the reader is left to follow. Suggest a general diagram, eg. Flow chart, decision tree to describe steps taken in the present analyses.

- **Manual queries and analyses are performed at several points in the workflow, which raise the question of validation and reproducibility. Subjective or inconsistent use of terms, for example, would invalidate findings. Suggest performing on >1 AOP to test level of reproducibility.**

This reviewer is concerned with the validity and consistence of gene-chemical vs. chemical phenotype groupings with various levels of confidence. Authors do not specify confidence level or thresholding for association groups. Same comment for “shared term percentage”-not reproducible with the current workflow explanations.

- **Concern with estrogenic effects being based only on ESR1, GENE:2099; ESR2, GENE:2100.** “Estrogen receptors regulate a multitude of biological and physiological processes” (Fuentes, 2019, doi: 10.1016/bs.apcsb.2019.01.001) Estrogenic effects occur via multiple mechanisms, pathways and receptors, some of which are not fully understood at present (Marino , 2006 doi: 10.2174/138920206779315737)
- **Supplemental training materials are suggested to facilitate reproducibility of the methods presented**
- **Process of manual mapping as presented could be improved (AOP Wiki KE terms to CTD phenotype).  **The standardization of mapping molecular identifiers to AOPs is an area of need in the AOP field. However, the Authors use term mapping from AOP-Wiki key events to map to CTD phenotype terms. This manual process could be improved for reproducibility and accuracy. Suggest implementing ontology-based gene mapping to CTD chemical-gene pairs and possibly using a publicly available AOP tool that provides this mapping, like the AOP-DB (Mortensen, Senn, 2021; Pittman, 2018); AOP-DB RDF (Mortensen, Martens, 2022) or AOP-WIKI EXPLORER (Saurav, 2024) , followed by mapping to ctd chem-gene or manually to phenotype. The AOP-DB ( Mortensen, Senn , 2021) actually maps AOP molecular KE (entrez genes ) directly to CTD-Gene tables, as well as DTXIDs, which could minimize the manual curation (and chance for error).

 

- **Please reference the literature and relevant contributions in this area **

*In Limitations and Strengths section*

-
  - **Suggest referencing the citations (Ives, et al 2017; Pittman, 2018; Mortensen, Senn 2021).** *“some biomedical data translators” are reference to assist in mapping KE to gene ontology terms”.  *
  - **Suggest referencing the extensive work in this area that preceded/contributed to the cited contributions (Pittman, 2018; Mortensen, Senn 2021; Mortensen, Martens, 2022) as well as other semantic mapping approaches (Saurav, 2024).** *“Other methods have been developed to increase the usability of AOP information, such as converting the AOP-Wiki into semantic web formats* *^58^* *or curating relevant KEs to gene sets associated with pathways, phenotypes, and GO terms* *^59^* *”. *

Is the work clearly and accurately presented and does it cite the current literature?

Partly

If applicable, is the statistical analysis and its interpretation appropriate?

I cannot comment. A qualified statistician is required.

Are all the source data underlying the results available to ensure full reproducibility?

Partly

Is the study design appropriate and is the work technically sound?

Partly

Are the conclusions drawn adequately supported by the results?

Partly

Are sufficient details of methods and analysis provided to allow replication by others?

Partly

Reviewer Expertise:

NA

I confirm that I have read this submission and believe that I have an appropriate level of expertise to confirm that it is of an acceptable scientific standard, however I have significant reservations, as outlined above.

# Response

**Published:** 2026-04-14

**Authors:** Allan Davis

**Affiliations:** CTD, USA

**1. Issue with selected AOP-Status “open for adoption”.**

**AUTHORS’ RESPONSE**: The intention of our manuscript was to show how researchers can leverage CTD in a variety of ways to help inform, refine, expand, and hopefully advance the status of underdeveloped AOPs.  When we initially submitted our manuscript (17 November 2025), the AOP-Wiki contained 532 AOPs, of which only 7% were designated as endorsed by WPHA/WNT, with the remaining 93% of the AOP-Wiki content described as empty (74%), under development (16%), under review (3%), and none with ESCA approval (0%).  In our minds, these statistics are indicative of a critical need for new methodologies to advance the status of these languishing AOPs as a way to improve the overall utility and resourcefulness of the AOP-Wiki.  The AOP used in our demonstration (i.e., AOP:522) is a good representative candidate, because, as this reviewer noted, it is “open for adoption”, currently poorly populated in the AOP-Wiki itself, and has equivocal limiting supporting evidence from the literature (although enough to warrant the construction of and deposition in the AOP-Wiki).  As Mari-Bauset et al. (2018) [rather than the mistakenly cited Mandy and Lai, 2016] conclude in their article about EDC and ASD: “incomplete understanding of biological mechanisms precludes the establishment of a causal relationship”, supporting the primary need to better understand the biological mechanistic steps that can link these disrupting chemicals to ASD, such as the development of a more robust AOP.  In our manuscript, we demonstrate how researchers can (1) leverage CTD to identify environmental chemicals that could modulate autism etiology via this AOP and (2) then use those identified chemicals to point researchers in mechanistically-supported directions to further develop and refine AOP:522 to a better position for testability and advancement.  To help clarify our intention, we have improved the text.  In response to similar comments from Reviewer 2, we have edited the last paragraph of the “Introduction” to now more clearly explain that our test AOP is underdeveloped, open for adoption, and currently contains limited data, and that by intersecting CTD content with this AOP, users can discover chemicals, genes, and phenotypes that can help develop and strengthen this AOP.

**2. Manuscript would benefit from an analysis workflow**

**AUTHORS’ RESPONSE**:  We have added a new comprehensive file of Supplemental Figures that includes a diagram of the overall approach and provide the step instructions and workflow for data acquisition and analyses, allowing for reproducibility of the methods presented.  This file is now available as an additional extended data set via the updated figshare link.

**3. Manual queries and analyses are performed at several points in the workflow, which raise the question of validation and reproducibility**

**AUTHORS’ RESPONSE**:  The crux of this work is to demonstrate how users can leverage CTD chemical content and readily integrate it with AOPs to (1) identify environmental chemicals that could potentially modulate autism etiology via interaction with AOP:522 and (2) then use these chemicals to discover new, potential mechanistic steps to further develop and refine AOP:522 to a better position for testability and advancement.  A step-by-step instruction guide is now provided (see new Supplemental Figures at figshare) for all the manual queries and analyses, enabling both validation and reproducibility.  However, it is important to understand that CTD is updated each month with new curated content from the recently published literature; this is one of the strengths of CTD. Thus, query results can change on a monthly basis (as currently explained in our “Methods” section).  Importantly, results from newer queries do not invalidate findings, but rather provide additional data from the more recent literature.  In Figure 2, we grouped the 76 prioritized chemicals simply as a display convenience (e.g., clustering together similar chemicals, such as metals, phthalates, pesticides, etc.).  The clustering itself does not impart any additional knowledge to the figure (compared to not clustering the chemicals).  We performed that task, as described in the manuscript, using web searches or shared term parentage in the CTD Chemical vocabulary hierarchy.  For example, “dibutyl phthalate”, “diethylhexyl phthalate”, and “monobutyl phthalate”, all share “Phthalic Acids” as a common parent in the CTD chemical hierarchy, and thus can be grouped as such.  Figure 2 is just as valid and reproducible if the chemicals were not clustered, and instead listed alphabetically.

**4. Concern with estrogenic effects being based only on ESR1, GENE:2099; ESR2, GENE:2100**

**AUTHORS’ RESPONSE**: In our manuscript, we did not base estrogenic effects only on the two estrogen genes (ESR1 and ESR2).  Rather, we mapped this MIE:112 to four CTD terms (Table 1): the two estrogen receptor genes ESR1 and ESR2 plus, importantly, two additional estrogen-signaling related phenotypes (which include all of their GO-phenotype descendants as well) to capture chemicals that still affect the estrogen receptor signaling pathway but without necessarily affecting the specific estrogen receptors.  It is important to understand that at CTD a chemical-phenotype interaction does not (necessarily) have to involve a gene, but is simply a curated annotation describing how a chemical causes a biological outcome reported in the literature; e.g., “chemical X results in decreased intracellular estrogen receptor signaling pathway in MCF-7 cells” is a chemical-phenotype interaction reported by an author without any discussion or knowledge of the genes involved.  This specifically enables CTD to capture literature-based chemical-induced outcomes without having to know a priori what genes are involved.  We would direct the reviewer to our detailed description of this CTD curation module to better understand what we mean as chemical-phenotype interactions (Davis et al., 2018). This CTD curation paradigm allows CTD to capture important data for chemical-induced events directly, without having to involve any genes (either known or unknown by the author).  Thus, chemicals that affect estrogen receptor signaling by “mechanisms which are not understood” (Marino, 2006) can still be captured in CTD because we curate the direct chemical-phenotype relationship itself, obviating the need to invoke any unknown gene/mechanism.  If a user chooses to broaden their interpretation of “estrogen receptor antagonism”, they can select a more expansive phenotype in the ontology by using a parent term, such as “steroid hormone mediated pathway” (GO:0043401), which now expands the query range and at this writing returns 231 unique chemicals instead of the original 180 chemicals shown in Table 1 for the more specific phenotype.  Curating phenotype data as an ontology (instead of as presumptive “gene set) is an advantage, and enables users to broaden or narrow their searches by navigating the vocabulary to the level they prefer.  If deemed necessary, users can always map this particular AOP step to additional genes beyond ESR1 and ESR2 if they prefer: this will only bring back additional chemical data for analysis.  Finally, a user could try to leverage Natural Language Processing (NLP)-based mapping tools that attempt to link KEs first to “gene lists” and then find CTD chemicals that interact with those gene sets.  NLP methods, however, are notoriously complex and difficult to follow and validate for the non-technical user, and, unfortunately, the results may go out of date quickly unless the NLP tool is updated on a consistent schedule; in fact, spot checks by CTD found some strange and equivocal mappings (discussed below) performed by such NLP methods.  The main point here, however, is that individual users have the ability to leverage CTD to their level of specificity, comfort, and skill level to (1) identify environmental chemicals that could potentially modulate autism etiology via interaction with this AOP and (2) then use these chemicals to discover new, potential mechanistic steps to further develop, expand, and improve the AOP to a better position for testability and advancement.  Different users might decide to use different terms to retrieve data, and that’s perfectly fine.  The user ultimately decides how large of a data-net he/she wants to cast and then can decide which molecular mechanisms retrieved from those results make the most sense to include in a testable updated/new AOP.  CTD provides the chemical data-set and evidence to fill in the mechanistic knowledge gaps connecting exposure to an adverse outcome.  To better reflect this, we edited all occurrences in the “Results and Discussion” of “This KE mapped to…” to the better phrase of “We mapped this KE to…” to emphasize that users are in control of the process here.

**5. Supplemental training materials are suggested to facilitate reproducibility of the methods presented.**

**AUTHORS’ RESPONSE**:  We have added a new comprehensive file of Supplemental Figures that includes a diagram of the overall approach and provide the step instructions and workflow for data acquisition and analyses, allowing for reproducibility of the methods presented.  This file is now available as an additional extended data set via the updated figshare link.

**6. Process of manual mapping as presented could be improved (AOP Wiki KE terms to CTD phenotype).**

**AUTHORS’ RESPONSE**: We wholeheartedly agree and promote the idea of the AOP research community coming together to diligently standardize their mappings, identification, and definitions.  We attempted to use many of the third party tools currently promoted by the AOP-Wiki in our original mapping and analysis, but unfortunately, we began to realize that many of these resources are unreliable, as they were either outdated, non-intuitive as to how to use, no longer available as a web application, or simply returned generic links back to the AOP-Wiki without providing any new context:

 AOP-DB (as defined by Mortensen, Senn, 2021; Pittman, 2018) is listed as a third-party tool on the AOP-Wiki, but is no longer accessible from its stated URL, and the EPA site appears to have been last updated May 2021 and also is inaccessible from its stated URL.

 Wiki Kaptis has a copyright stamp of 2022, and the information menu states that it only contains data extracted from the AOP-Wiki up to 2023.  Our searches using official AOP-Wiki terms such as: AOP:522, autism (AO:2209), estrogen receptor antagonism (MIE:112), ERK1/2 inhibition (KE:2207), and aberrant synaptic formation and plasticity (KE:2208) yielded no results.

 The AOP-KB is a tool provided by the OECD, but again, appears to be limited in functionality.  A user can enter any term (e.g., “estrogen receptor”) and retrieve term matches to AOPs and KEs such as “Androgen receptor activation leading to prostate cancer”, but this result simply links back to the AOP-Wiki, with no explanation as to why this AOP would be returned as a result for “estrogen receptor”.  It is not obvious how the AOP-KB determined that “estrogen receptor” is involved in this AOP.  Similarly, a search with “autism” returns no results, suggesting the AOP-KB is also not current and out-of-date with the AOP-Wiki.

 AOP-WIKI EXPLORER (as defined by Kumar et al. 2024) is no longer accessible from its stated URL.

 We next attempted to use a tool that employs NLP to map curated genes to KEs (Saarimaki et al. (2023), but the resource also seems to be out of date, and spot-checks performed by us gave questionable results: e.g., MIE:112 (“estrogen receptor antagonism”) was mapped to 59 genes, but surprisingly did not include ESR2, the second critical estrogen receptor in humans; similarly, KE:195 (“NMDARs inhibition”) contained only seven GRIN genes, missing 16 other GRIN genes that we included in our CTD analysis.  The other KEs we checked (KE:2207, KE:2208, KE:2209) do not even exist in the file, presumably because of its outdatedness.

 Based upon these unsatisfying and inconsistent approaches, in this manuscript, we decided to map the six AOP terms to CTD genes and phenotypes manually ourselves, as a typical CTD user would find it necessary to do.  Going forward, it would be more advantageous if the AOP-Wiki recommended tools that are more easily and readily accessible, stable, intuitive to use, and designed for data currency, otherwise they risk becoming out of date with diminishing value as time goes on.

**7. Please reference the literature and relevant contributions in this area.**

**AUTHORS’ RESPONSE**:  Thank you.  To the “Limitations and Strengths” section, we have now added citations for: Ives et al. (2017): Creating a Structured AOP Knowledgebase via Ontology-Based Annotations; Pittman et al. (2018): AOP-DB: A database resource for the exploration of Adverse Outcome Pathways through integrated association networks; Mortensen et al. (2021): The 2021 update of the EPA’s adverse outcome pathway database; and Kumar et al. (2024): AOPWIKI-EXPLORER: An interactive graph-based query engine leveraging large language models.
